# Profile of suicide within the northern part of Ghana: A decade under review

**DOI:** 10.4102/sajpsychiatry.v28i0.1620

**Published:** 2022-01-27

**Authors:** Paul P.S. Ossei, Nicholas Niako, William G. Ayibor, Emmanuel Asante, Foster K. Safo, Adwoa Safowaa

**Affiliations:** 1 Department of Pathology, KSMD, Kwame Nkrumah University of Science and Technology, Kumasi, Ghana; 2 Department of Pathology, Komfo Anokye Teaching Hospital, Kumasi, Ghana; 3 Department of Molecular Medicine, KSMD, Kwame Nkrumah University of Science and Technology, Kumasi, Ghana; 4 Department of Epidemiology and Biostatistics, Kwame Nkrumah University of Science and Technology, Kumasi, Ghana

**Keywords:** medico-legal, suicide, methods of suicide, poisoning, hanging

## Abstract

**Background:**

Several reports show that suicide is the second and third leading cause of untimely death in young people below the age of 30. Little, however, is known about the profile and trend of suicide in this country due to lack of systematic studies and a lack of national statistics on suicide. This study seeks to examine the profile and pattern of suicide cases recorded within northern Ghana for the past decade.

**Aim:**

This study aimed to report the prevalence of suicide as an independent cause of death; the choice of suicide method and the alleged reasons for suicide within the northern part of Ghana.

**Setting:**

Retrospective review of coroners’ reports within the northern part of Ghana.

**Method:**

In this descriptive study, 309 completed suicides as archived by the office of the coroner were examined. The coroners’ reports of 309 individuals, whose deaths received a suicide verdict or an open verdict in which the cause of death was likely to be suicide from 2008 to 2017, were examined. Student’s t-test was used to ascertain significant age differences between the genders involved.

**Results:**

Amongst the 309 decedents examined, approximately, 61% were male, with ages ranging from 5 to 81 years. Hanging and poisoning were the most commonly used methods to complete suicide accounting for 124 (40.1%) and 102 (33.0%) deaths, respectively. Regarding the reasons for completed suicide, 78 (25.2%) were because of unknown reasons and 66 (21.4%) were because of social stigma. There was a notable decline in the prevalence of suicide from 2014 to 2017 compared with the years from 2010 to 2013.

**Conclusion:**

Suicide was highest in the 30–39 year age group with hanging and poisoning being the most common method employed. Stigmatisation and psychosocial problems arising from chronic illness and economic hardship were significant triggers of suicide amongst the suicide decedents in the northern part of Ghana.

## Introduction

The global phenomenon of suicide is disconcerting. Reports show that suicide is the second and third leading cause of untimely death in persons between the ages of 15 to 29 (preceded only by road accidents in this category) and 15 to 44 years age groups, respectively.^1^ The statistics of suicidal deaths are sobering. According to the World Health Organization (WHO), nearly 1 000 000 people died from suicide in the year 2000 alone. It is further estimated that close to 800 000 people die of suicide every year and that for each completed suicide there are more than 20 attempts with a global mortality rate of 16 per 100 000 people. Put differently, these estimates portend that someone will commit suicide every 40 s each year.^2,3^ Sadly, these statistics have served as a painful barometer of the ongoing universal public health problem.

Amongst the reasons for completed suicide, studies on suicide ideations show that suicide has been the manner of death of many who suffer from depression,^4^ substance abuse, mood disorders, schizophrenia and other socioeconomic crisis such as debt, unemployment and other unpleasant conditions in which most live each day.^5,6,7^

According to the 1960 *Criminal Code Act* 29, Section 57 of Ghana, attempted suicide is considered a crime.^8^

Alluding to this, the WHO has encouraged countries such as Ghana (who according to situational analysis has close to 650 000 people suffering from a severe mental disorder and another 2 166 000 suffering from a moderate to mild mental disorder^9^) to decriminalise it because suicidal behaviours are associated with mental health issues and that people who attempt to commit suicide should rather be helped and not prosecuted.^10^ In Ghana, some studies have listed loss of economic control, hopelessness and sexual incompetence amongst others as prominent causes of suicide.^11,12^ Despite these reports, there are inadequate systematic studies and a lack of national statistics on suicide, thus little is known about the profile and trend of suicide in the country. This study, therefore, aims to examine the profile and pattern of suicide cases recorded within northern Ghana for the past decade from 2008 to 2017.

## Method and subjects

The data for this study were abstracted from the coroners’ case records. Details of all inquests on people who had died between January 2008 and December 2017 in which a verdict of suicide or likely suicide was made, were retrieved from coroner’s offices within the northern part (Ashanti region, Brong-Ahafo Region, and some parts of the Savannah region) of Ghana. Together, the study area covers nearly 42.0% of Ghana’s total land surface, with more than 8.9 million inhabitants as of 2019.^13^ With nearly 4.6 million and 4.3 million females and males, respectively. During the study period, there were 7846 medico-legal cases from the listed Coroner’s offices, of which 309 (3.94%) had the suicide verdict.

### Variables

Methods of suicide as stated by the International Classification of Disease version 10 (ICD-100)^14^ were divided into six groups: (1) hanging (which includes strangulation and suffocation) (X70), (2) poisoning by drugs (X60–X64), (3) poisoning by other means (X66–X69), (4) firearms and explosives (X72–X75); (5) Jumping from height (X80) and (6) other methods (X71, X76–X79, X81–X84).

Demographic details, mode of suicide (firearm, poisoning, hanging, etc.) and circumstances leading to the death as documented in the statements for the coroner of relatives, police or witnesses were accessed for the study.

Within the reports, witness accounts of discrimination or prejudiced attitudes directed at individuals with certain health or societal labels were classed as stigmatisation. Reports having a missing or incomplete record of the alleged cause of suicide were classified as not recorded in this study. The data were organised and analysed using Microsoft Excel and GraphPad Prism 8.

## Results

The study showed a mean and median age of 34 years 99 days (34.27 years) and 32 years, respectively, ranging from 5 to 81 years. Most of the suicide victims were under the age of 39 (68.5%) years, with the age group 30–39 years recording the highest proportion of cases 91 (29.4%). Males were the predominant victims, accounting for 188 (60.8%) of the cases with a ratio of 1.55:1 female. The 309 cases of suicidal deaths represent a prevalence rate of 3.94% in northern Ghana. Hanging accounted for the most common means of suicide 124 (40.1%) followed by poisoning 99 (32.0%) of all the cases. Collectively these two most common means of suicide represents 71.8% of all cases within the 10 years. The results are summarised in Table 1.

**TABLE 1:** Age and sex distribution of decedents.

Variable	Sex		*n*	%
	Female	Male		
**Age distribution**				
< 10 years	4	2	6	1.9
10–19 years	25	22	47	15.2
20–29 years	31	37	68	22.0
30–39 years	39	52	91	29.4
40–49 years	13	40	53	17.2
50–59 years	6	13	19	6.1
60–69 years	3	12	15	4.9
70–79 years	-	8	8	2.6
>79 years	-	2	2	0.6

**Total**	**121**	**188**	**309**	**100.0**

Note: mean is 29.93 (female) and 37.06 (male). Standard deviation is 12.4 (female) and 16.0 (male). *P*-value: < 0.001. Median is 30 (female) and 35 (male). Range is 5–65 years (female) and 7–81 years (male).

**FIGURE 1:**
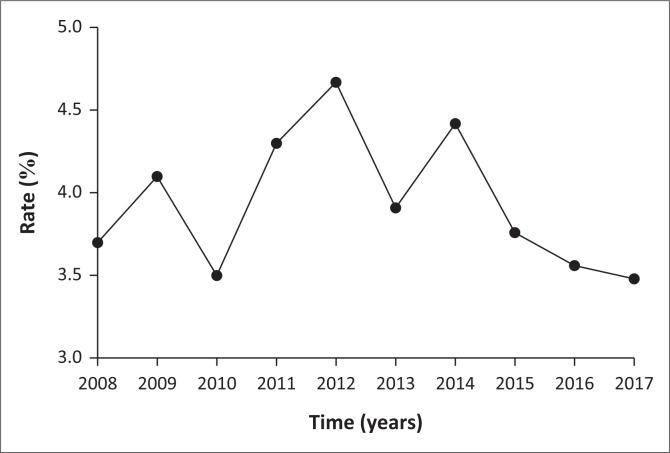
Trend of suicide prevalence in all medico-legal cases examined over the decade 2008–2017.

The yearly trend of suicide prevalence rates for the decade in the review is shown in Figure 1. The year 2012 recorded the highest rate (4.67%) of suicides of all medico-legal cases within that year, followed by 4.42% in 2014, 4.30% for 2011 and 4.10% in 2009, 3.91% in 2013, 3.76% in 2015, 3.70% in 2008, 3.56%, 3.50% and 3.48% in the years 2016, 2010 and 2017, respectively.

As shown in Table 3, 78 (25.2%) of all the suicides were because of unknown causes or had missing information, 66 (21.4%) were allegedly because of stigmatisation followed by debilitating chronic illnesses suffered by victims 64 (20.7%). Economic hardship accounted for 37 (12.0%) and escape from prosecution 35 (11.3%) in that order as shown in Table 3.

## Discussion

In our study, 60.8% of suicide decedents were male. This gender disparity is consistent with results from wider studies conducted in the United States of America, which found that nearly 77% of all suicide deaths occur amongst men.^15,16^

**FIGURE 2:**
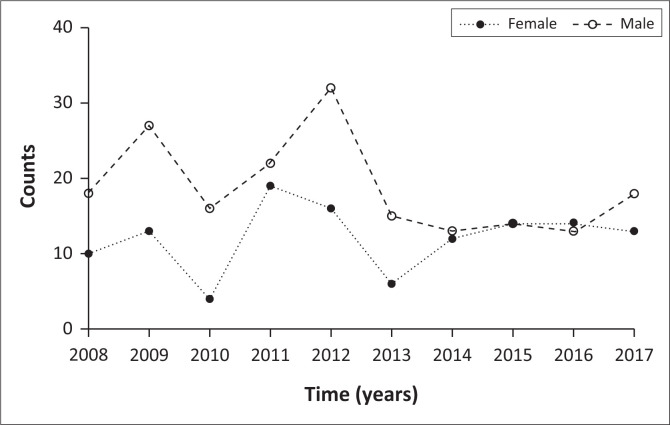
Trend of suicide by gender for the 2008–2017 decade.

Similarly, Kolves et al.^17^ and Keugoung et al.^18^ showed that 77% and 78% of all completed suicide victims in Australia and Cameroon, respectively, were male. Furthermore, the trend of suicide amongst females had stayed lower compared with their male counterparts throughout the decade except for years 2015–2016 as depicted in Figure 2. Thus, Figure 2 highlights the higher risk of suicide amongst males.

**FIGURE 3:**
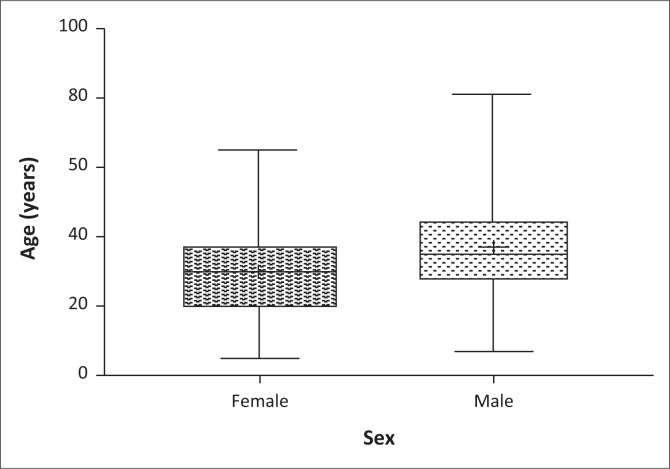
Age distribution of suicide decedents amongst males and females.

Table 1 shows the age-related rates of suicide cases. The rates show an increase from adolescence peaking at ages 30–39, with a gradual decline to old age. Suicide amongst adolescents has shown an upward trend as observed by Quarshie et al.^19^ who in their content analysis of 44 suicide cases from 2001 to 2014 revealed 40 (90.9%) completed suicide with an average age of 15.4 and 16 years for females and males, respectively. Sadly, official statistics on suicide are lacking that makes it difficult to ascertain the national pattern of suicide amongst age groups in Ghana. Notwithstanding, women in this study appeared to be younger on average, with a mean age (standard deviation [s.d.]) of 29.9 (12.4) years and a median age of 30 years versus average age (s.d.) of 37.1 (16.0) years and a median age of 35 years between men (Figure 3), this disparity was statistically significant (*p* < 0.0001) Table 1.

From our results, hanging (which include suffocation) accounted for 124 (40.1%) of all the cases followed by poisoning in 102 (33.0%) Table 2. Collectively these two methods of suicide represented 72.1% of all deaths. Whilst the prevalence of suicide methods, varies by country, hanging remains the most common method of suicide for both sexes even in high-income countries^3^ except the United States of America where firearms account for nearly 51% of all suicides^20,21^ in contrast to 8.7% as observed in the present study.

**TABLE 2:** Mode of suicide by gender.

Mode of suicide	Sex		*n*	%
	Female	Male		
Hanging	50	74	124	40.1
Poisoning	54	48	102	33.0
Firearm	3	24	27	8.7
Jumped from height	5	12	17	5.5
Electrocution	3	13	16	5.2
Drowning	4	6	10	3.2
Slitting/stabbing	1	8	9	2.9
Ran-into-car	1	2	3	1.0
Run-into-wall	-	1	1	0.3

**Total**	**121**	**188**	**309**	**100.0**

Poisoning, mainly by ingestion of agrochemicals (organophosphates), is reckoned amongst the most common means of suicide in Asia^22^ and other agricultural rural regions of the world,^18,20^ where agrochemicals such as pesticides and weedicides are easily accessible. Existing literature suggests that poisoning by agrochemicals accounts for nearly 30% of global suicides and this method is the most common means of suicide in lower and middle-income countries (LMICs).^3^ Thus, the high percentage of suicide because of poisoning recorded in our study agrees with this.

Present studies have shown that jumping from a height as a method of suicide is very common in highly urbanised cities such as Singapore and Hong Kong^23,24^ This is contrasted by a rather lower number of cases (17 [5.5%]) in this study, with 5 men and 12 women, respectively (Table 2). Eleven of those who jumped from height occurred in metropolitan areas. This finding is coherent with Kõlves et al.^17^ who also showed that people residing in rural and remote areas and those experiencing interpersonal conflict were less likely to commit suicide by jumping from height relative to other methods. Their work showed that people who were born overseas or had mental illnesses were more likely to jump and are less likely to act under the influence of alcohol.

Electrocution is a rare mode of suicide, and deaths due to electrocution from the domestic supply of 220 volts are usually due to accidents. However, in this report, 16 (5.2%) of the cases had the verdict of suicide. whereas the medical records of these numbers were inaccessible for the study, Fernando et al.^25^ in his work reported a 34-year-old man who committed suicide by electrocution. His medical history showed that he suffered depressive illness although there was no family history of psychiatric illness. Amongst others, drowning, stabbing/slitting, inter alia (Table 2) collectively represent 7.4% of all suicide methods.

The motivation or reason for committing suicide is enigmatic and variable. Our analysis showed that 78 (25.2%) of the cases were for unknown reasons. Another 66 (21.4%) were tied to stigmatisation. This is consistent with other Ghanaian studies.^12,26^ Thus, psychosocial strains significantly contribute to suicide in Ghana, a pattern noticeable in many LMIC.^27,28^ Next on the list of causes, 64 (20.7%) of the cases were because of chronic illnesses suffered by decedents. Chronic diseases are disorders that are not cured by medical treatment and require routine monitoring and management to alleviate the severity of the disease.^29^

Chronic diseases are a major source of stress because of the effects they have on the individual’s life; disrupting his or her harmony of life, symptom-related problems, loss of certain physical abilities and the treatment process alone coupled with financial constraints can trigger suicidal behaviours. As reported by Misono et al.^30^ patients diagnosed with cancer have a higher risk of committing suicide. Similarly, in individuals with diabetes mellitus, Pompei et al.^31^ discovered that poor quality of life was linked to low self-efficacy, excessive despondency and suicidal tendencies.

On the other hand, Economic hardship accounted for 37 (12.0%). Another 35 (11.0%) chose to escape arrest/prosecution by committing suicide. Disappointment (mainly from failed relationships), abuse (sexual, physical and verbal), depression and loneliness collectively were the cause of 9.4% of suicide cases in this study as shown in Table 3. Whilst these findings are upsetting, solace can be taken from the trend of suicide prevalence amongst medico-legal cases within the decade. Suicide prevalence appears to be decreasing since the year 2015 after reaching a peak in the year 2012 (Figure 1).

**TABLE 3:** Alleged circumstance or reason for committing suicide.

Cause/circumstance	Total	(%)	Cummulative percentage (%)
**Not recorded**	**78**	**25.2**	**25.2**
**Stigmatisation**	**66**	**21.4**	**46.6**
Infertility/impotency	19	28.8	-
Accused of witchcraft	18	27.3	-
Accused of theft	10	15.2	-
Academic failure	8	12.1	-
Unwanted pregnancy	7	10.6	-
Accused of infidelity	2	3.0	-
Accused of murder	2	3.0	-
**Chronic disease**	**64**	**20.7**	**67.3**
HIV and/or AIDS	25	39.1	-
Cancer	20	31.3	-
Epilepsy	7	10.9	-
Others	12	18.8	-
**Economic hardship**	**37**	**12.0**	**79.3**
Debt	21	56.8	-
Unemployment	14	37.8	-
Loss of fortune	2	5.4	-
**Escape arrest/prosecution**	**35**	**11.3**	**90.6**
**Disappointment**	**12**	**3.9**	**94.5**
**Abuse**	**10**	**3.2**	**97.7**
Child abuse/physical	6	50	-
Sexual	5	41.7	-
Verbal abuse	1	8.3	-
**Mental/depressive illness**	**4**	**1.3**	**99.0**
**Loneliness/negligence**	**3**	**1**	**100**

**Total**	**309**	**100.0**	**-**

HIV, human immunodeficiency virus; AIDS, acquired immunodeficiency syndrome.

In this study, the mental health aspects (such as depressive illness and schizophrenia amongst others) of the suicide decedents could not be ascertained from chart abstractions or the coroners’ reports. Thus, research on uncompleted or attempted suicides on whom mental health assessments can be made is highly recommended.

## Conclusion

Suicide rates were highest among those aged 30–39 years, with hanging and poisoning being the domineering methods of suicide among the residents of Northern Ghana. Amongst the reasons for committing suicide, nearly one-fourth of all suicides were because of unknown causes, the remaining three-fourths of the cases were as a result of social stigma, chronic or debilitating illness, economic hardship and other psychosocial factors.
